# Internet of things (IoT) for safety and efficiency in construction building site operations

**DOI:** 10.1038/s41598-024-78931-0

**Published:** 2024-11-21

**Authors:** Abdul Mateen Khan, Khaled A. Alrasheed, Ahsan Waqar, Hamad Almujibah, Omrane Benjeddou

**Affiliations:** 1 Department of Civil Engineering, International Islamic University, Islamabad, 44000 Pakistan; 2 College of Engineering and Petroleum, Kuwait University, Kuwait City, Kuwait; 3 University of Lahore, Gujrat Campus, Jalalpur Jattan Road, Gujrat, Punjab 50700 Pakistan; 4 Department of Civil Engineering, College of Engineering, Taif University, P.O. Box 11099, Taif City, 21974 Saudi Arabia; 5 Department of Civil Engineering, College of Engineering, Prince Sattam bin Abdulaziz University, Alkharj, 16273 Saudi Arabia

**Keywords:** Environmental monitoring, Equipment management, Predictive analytics and maintenance, Safety monitoring, Engineering, Civil engineering

## Abstract

Internet of Things (IoT) technologies present transformative opportunities through connectivity of intelligent devices, environmental sensors, and integrated management systems. This study aims to investigate the benefits and impact of IoT implementation on construction sites by analyzing relationships between key factors and outcomes for safety and efficiency. Hypotheses were developed proposing positive correlations between each factor and effective IoT adoption on construction sites. Structural equation modeling analysis on survey data from construction professionals and site reports strongly validated the research hypotheses. Positive path coefficients and high statistical significance confirmed environmental monitoring (0.38), equipment management (0.343), predictive analytics and maintenance (0.222) and safety monitoring (0.369) as crucial enablers for successful IoT integration leading to safer and more productive construction operations. The findings highlight imperative focus areas and provide actionable insights for construction stakeholders on strategies to effectively leverage IoT capabilities.

**Supplementary Information:**

The online version contains supplementary material available at 10.1038/s41598-024-78931-0.

## Introduction

The dynamic construction industry constantly seeks to improve operational efficiency and enhance safety in hazardous work environments^1,2^. Recent advances on the Internet of Things (IoT) enable connectivity between intelligent devices, sensors, and systems through wireless networks. Implementation of IoT technologies presents an unprecedented opportunity to introduce data-driven real-time monitoring and decision-making onto construction sites^3,4^. This integrated approach has the potential to revolutionize safety practices and unlock significant productivity gains on construction projects^5,6^. Early case studies demonstrate the transformative impact IoT can have. Predictive maintenance powered by equipment sensor data has reduced downtime incidents by 25–30% and improved asset utilization by 10–15%^7,8^. Similarly, wearable safety monitoring systems for workers have correlated with 40% reductions in accidents and injuries across multiple sites^9,10^. Environmental sensing and analysis of air quality, noise levels and other hazards have shown a 15% drop in work-related illnesses, directly contributing to safer conditions^11,12^. Despite the clear benefits, comprehensive studies examining large-scale IoT implementation specifically for construction sites remain limited. Research has predominantly been fragmented across application areas without holistic understanding of adoption drivers, challenges and best practices contextualized for the unique requirements of the construction industry. In particular, safety improvements represent one of the greatest untapped opportunities, as construction remains one of the most hazardous sectors globally^13,14^. Existing literature lacks in-depth examination of how emerging IoT technologies like wearables, real-time monitoring systems, and wireless sensor networks can enhance identification and mitigation of worksite hazards to reduce incidents and near-misses^15,16^. The proper implementation of IoT technologies has immense transformative potential for construction projects. However, real-world deployments at scale remain nascent. There is a pressing need for rigorous academic research and industry collaboration to develop effective strategies and best practices tailored to construction’s unique operating environment^17,18^. While IoT adoption has been transformative across many industries, construction projects continue to face challenges like cost and schedule overruns, resource wastage, and poor safety records^4,19^. Comprehensive data-driven solutions enabled by IoT integration hold immense potential to optimize construction performance through real-time monitoring, predictive analytics, and automated workflows^16,20^. However, more research is critically needed to understand how IoT technologies can be most effectively leveraged for construction site management. Key untapped opportunities exist in using IoT systems to realize significant cost savings, radically improve productivity rates, and dramatically reduce project timelines^21,22^. This necessitates in-depth investigation of IoT use cases, benefits quantification, implementation strategies, and impact assessment to inform decision-making in construction firms^23,24^. The aim of this present research study is to provide valuable and actionable insights for the industry by comprehensively analyzing the state of IoT adoption, evaluating demonstrated results on productivity and safety metrics, identifying potential barriers and risks, as well as assessing the business case and environmental impact. Focusing on construction activities in the developing economy context of Pakistan—this research explores the critical yet under-studied area of how pervasive IoT integration affects safety standards and operational efficiency metrics on building project sites. The Mirpur region of Pakistan was selected for this study due to its significant construction activity and rapid urban development, making it an ideal location for examining the impact of IoT on construction safety and efficiency. The region’s diverse construction projects and varied site conditions provide a comprehensive setting for analyzing IoT implementation. Additionally, focusing on a developing region like Mirpur offers valuable insights into the challenges and opportunities of IoT adoption in contexts with similar economic and infrastructural conditions. This makes the findings more relevant and applicable to other developing regions facing analogous challenges. While interest in smart construction is rapidly growing, current deployments have been limited to developing regions that can benefit tremendously from catalytic productivity and safety improvements. Through a rigorous structural equation modelling approach, this study aims to bridge the knowledge gap and establish an analytical framework for understanding the complex interrelationships between IoT system usage, resulting safety outcomes, and operating performance impacts across construction processes.

The Introduction provides background and context for the study, including the selection of the Mirpur region. The Literature Review covers previous research on IoT in construction and sets the stage for the current study. The Methodology section details the research design, data collection, and analysis methods. The Results section presents the findings of the study, followed by the Discussion, which interprets these findings in the context of existing literature. The Conclusion summarizes the key insights and implications of the study, and the References list all sources cited in the manuscript.

## Literature review

The construction industry has been slow to adopt new technologies compared to other fields, but the advent of the Internet of Things (IoT) is offering new opportunities to transform traditional construction sites in terms of safety, efficiency, sustainability, and management^25,26^. Recent literature has explored various promising applications of IoT across all stages of construction projects^24,27^. Smart helmets equipped with sensors, cameras and communication systems can continuously monitor workers, providing biometric data to assess health and fatigue levels in real-time^28,29^. They also enable improved safety by detecting potential hazards in the worker’s line of sight and communicating warnings or shutdown commands to vehicles and equipment in the vicinity. Exoskeletons and other body-worn devices provide muscular strength augmentation and injury prevention while gathering data on biomechanics, productivity, and training needs^30,31^. Handheld devices and augmented reality (AR) headsets allow contextual data gathering and access to 3D visualization overlaid on the workspace, improving work quality, training, and collaboration^32,33^. Location tracking of vehicles and equipment via GPS and sensors has also seen significant IoT integration on construction sites. Real-time positioning facilitates coordination of vehicle movements, significantly reducing the risk of collisions and resulting injuries, fatalities, and costs^34,35^. Sensor data enables optimized routing, scheduling, and utilization of machinery. Predictive maintenance reduces equipment downtime and costs through continuous monitoring of usage, vibrations, and other diagnostic metrics^36,37^. Automated tracking systems can also integrate with building information modeling (BIM) and schedule data for advanced analytics, providing rich insights into fleet management, bottlenecks, and process improvements^38,39^.

Recent literature highlights the immense potential of IoT technologies to transform safety, productivity, sustainability, compliance, and management on construction sites^10,40^. The expanded table provides a comprehensive overview of the diverse range of IoT applications and their benefits, while also noting key challenges that must be overcome as shown in Table 1. A major application area is monitoring worker safety and productivity through wearables and handheld devices^41,42^. These provide real-time visibility into health metrics, fatigue levels, training needs, work quality and collaboration effectiveness. Location tracking of both workers and equipment enables better coordination on congested sites, reduced accidents, and optimized workflows. Sensors on equipment and vehicles allow usage monitoring for preventive maintenance, routing and dispatch optimization, and reduction in collisions^43,44^. Thermal cameras, lidar and other structural health monitoring technologies facilitate early detection of cracks, deformation, and failures. Gas detection and air quality sensors ensure compliance with safety regulations and help prevent explosions, leakage incidents or worker health impacts from dust and emissions. Noise monitoring enables noise level management and hearing loss prevention^42,45^. Weather tracking allows proactive planning for work disruptions. Scanners, photogrammetry, and computer vision techniques validate construction quality and progress against specifications^22,46^. Data security and integration technologies are critical enablers, allowing aggregation of data across diverse systems for unified insights while protecting against cyberthreats^39,47^. Change management and training must also be addressed to drive adoption across the workforce. Compliance sensors ease regulatory reporting burden in areas like sustainability and worker exposures^35,48^. Technologies for materials tracking and automated reporting of progress metrics offer further opportunities to enhance construction site operations^49,50^. While the technologies hold great promise, challenges remain in areas like reliability, interoperability, power, calibration, interference, cost, manual oversight needs and technology limitations for specific use cases. However, the convergence of technologies like cloud computing, 5G connectivity, AI/ML analytics and robotic automation will help overcome many of these barriers^18,51^.

**Table 1:** Internet of things applications for next-generation construction sites.

Factor	Monitoring technology	Key data points	Benefits	Challenges	Metrics	Use Cases	Value drivers	References
Worker Safety	Smart helmets, wearables	Health metrics, fatigue, hazards	Injury prevention, risk alerts	Adoption, cost	Incident rates, unsafe behaviors	Gas alerts, fall detection	Reduced injuries, insurance	^52,53^
Worker Productivity	Handheld devices, AR/VR	Work quality, training needs	Enhanced tracking, training	Learning curve, adoption	Work volumes, rework rates	Workflow instructions, 3D visualization	Optimized labor, quality	^54,55^
Worker Location	GPS, BLE beacons	Movement, proximity alerts	Coordinate workers, avoid accidents	Privacy concerns, failures	Utilization rates, transit times	Proximity alerts, location visualization	Efficient workflows, space utilization	^56,57^
Equipment Location	GPS, RFID tags	Real-time location, zones	Optimize routes, prevent collisions	Interference, power	Downtime, transit and wait times	Traffic coordination, geofencing	Reduced damage, efficient use	^58,59^
Equipment Usage	Sensors, meters	Load, runtime, diagnostics	Predictive maintenance, reduce failures	Installation, data management	Sensor trends, abnormal events	Condition monitoring, utilization patterns	Lower maintenance costs, uptime	^60,61^
Vehicle Location	GPS, proximity sensors	Location, speed, proximity	Avoid collisions, optimize traffic	Cost, data integration	Accident rates, traffic violations	Automated dispatch, congestion alerts	Improved safety, fuel savings	^62,63^
Structural Health	Thermal cameras, lidar, gauges	Cracks, deformation, stresses	Early failure detection, prevent collapses	Technology limits, expert analysis	Crack growth, deformation trends	Walls, foundations, temporary structures	Avoid failures, quality	^64,65^
Gas Detection	Air quality sensors	Methane, CO, VOCs	Prevent explosions, leakage incidents	Maintenance, calibration	Gas concentration trends	Fuel storage, underground work	Compliance, worker safety	^66,67^
Noise Levels	Sound sensors, microphones	Decibel levels, frequencies	Prevent hearing damage, noise control	Interference, placement	Time over exposure limits	High noise areas, equipment	Compliance, health	^68,69^
Air Quality	Particle sensors	PM2.5/PM10, dust	Protect worker health, reduce hazards	Sensor reliability, accuracy	Pollutant concentration trends	Material storage, mixing stations	Compliance, health	^70,71^
Weather Tracking	Anemometers, humidity sensors	Wind, precipitation, visibility	Plan for weather disruptions	Costs, data integration	Work stoppages, weather delays	Wind-sensitive tasks, concrete curing	Avoid weather delays	^72,73^
Project Quality	Scanners, photogrammetry	Model validation, progress metrics	Ensure specifications met, identify defects	Expert analysis, data volume	Defect rates, rework rates	Structural inspection, progress tracking	Prevent rework, meet specifications	^74,75^
Data Security	Encryption, access controls	Protect against data theft, manipulation	Ensure safety, prevent fraud	Complex systems, auditing	Unauthorized access events, anomalies	Sensor data, personal information	Privacy, data integrity	^76,77^
Data Integration	APIs, standard protocols	Aggregate data across systems	Unified insights and analytics	Interoperability, legacy systems	Data coverage, gaps	Centralized analytics, visualizations	Improved decisions, automation	^78,79^
Compliance	Checklists, sensor data as proof	Adhere to regulations, demonstrate compliance	Avoid fines, business continuity	Manual effort, unclear requirements	Audit results, noncompliance events	Emissions, worker exposure levels	Avoid fines, prosecutions	^80,81^
Sustainability	Energy, water, waste sensors	Resource usage, carbon emissions	Improve environmental impact	Costs, data management	Energy/water/waste usage, carbon emissions	Equipment utilization, material sourcing	Meet certification goals, stewardship	^82,83^
Materials Management	Barcodes, RFID	Materials tracking, inventory	Improve supply chain visibility	Costs, adoption across supply chain	Stockouts, wastage	Delivery tracking, inventory management	Optimal material flow, minimized waste	^84,85^
Schedule Management	Work progress sensors, video analytics	Construction progress metrics	Identify delays, optimize schedules	Technology limitations, manual oversight still required	Schedule variance, milestone achievement rates	Automated punch list, work sequencing	On-time delivery, cost control	^86,87^
Automated Reporting	Wearables, equipment sensors	Activity tracking, output metrics	Automate paper/spreadsheet reporting	Adoption, ensuring data validity	Time savings, report automation rates	Daily logs, sensor-generated reports	Accelerated and consistent reporting	^88,89^
Remote Operations	Cameras, sensors, wearables	Site monitoring, controlling equipment remotely	Continue work during crises, harsh conditions	Legal limitations, tasks not suited for remote work	Percentage of work done remotely	Hazardous tasks, controlling machinery	Improved safety, continuity	^90,91^

Environmental monitoring using IoT-enabled sensors allows construction firms to ensure worksite safety, regulatory compliance, and achievement of sustainability targets^92,93^. Air quality sensors can detect hazardous gases, while particulate matter monitoring protects workers from dust inhalation risks^94,95^. Acoustic sensors facilitate noise level management to prevent hearing loss. Thermal imaging and other camera systems enable remote structural health monitoring and early detection of failures or weaknesses^96^. Meteorological sensors and forecast data integration allows for proactive planning and mitigation of weather disruptions. While the literature highlights the challenges of technical complexity, interoperability, cybersecurity and change management with large-scale IoT implementation, it is clear that IoT adoption has become essential for construction firms to gain operational efficiency, comply with regulations, achieve sustainability goals, and maintain a competitive advantage^97^. The consensus view is that the benefits far outweigh the costs, and construction companies that fail to innovate with IoT technologies will lag behind those leveraging real-time data and analytics to optimize safety, productivity, quality, and resilience on their sites^98^.

In conclusion, the literature comprehensively highlights the tremendous potential of Internet of Things technologies to transform safety, productivity, compliance, sustainability, and management on construction sites. From wearables for workers to equipment sensors, air quality monitors to structural inspection drones, the possibilities are immense. While challenges like costs, interoperability, cybersecurity, and technology limitations exist, the trends point toward IoT becoming an essential enabler for construction firms to gain competitive advantage through real-time visibility, data-driven insights, and cross-system integration. Companies that fail to sufficiently invest in IoT risk falling behind industry leaders who are aggressively leveraging the power of data. Construction enterprises must therefore focus on building robust IoT infrastructure and analytics capabilities to meet the demands of modern infrastructure projects. The future points toward smarter, safer, and more efficient construction sites enabled by the proliferation of connected technologies.

### Research framework

**Fig. 1:**
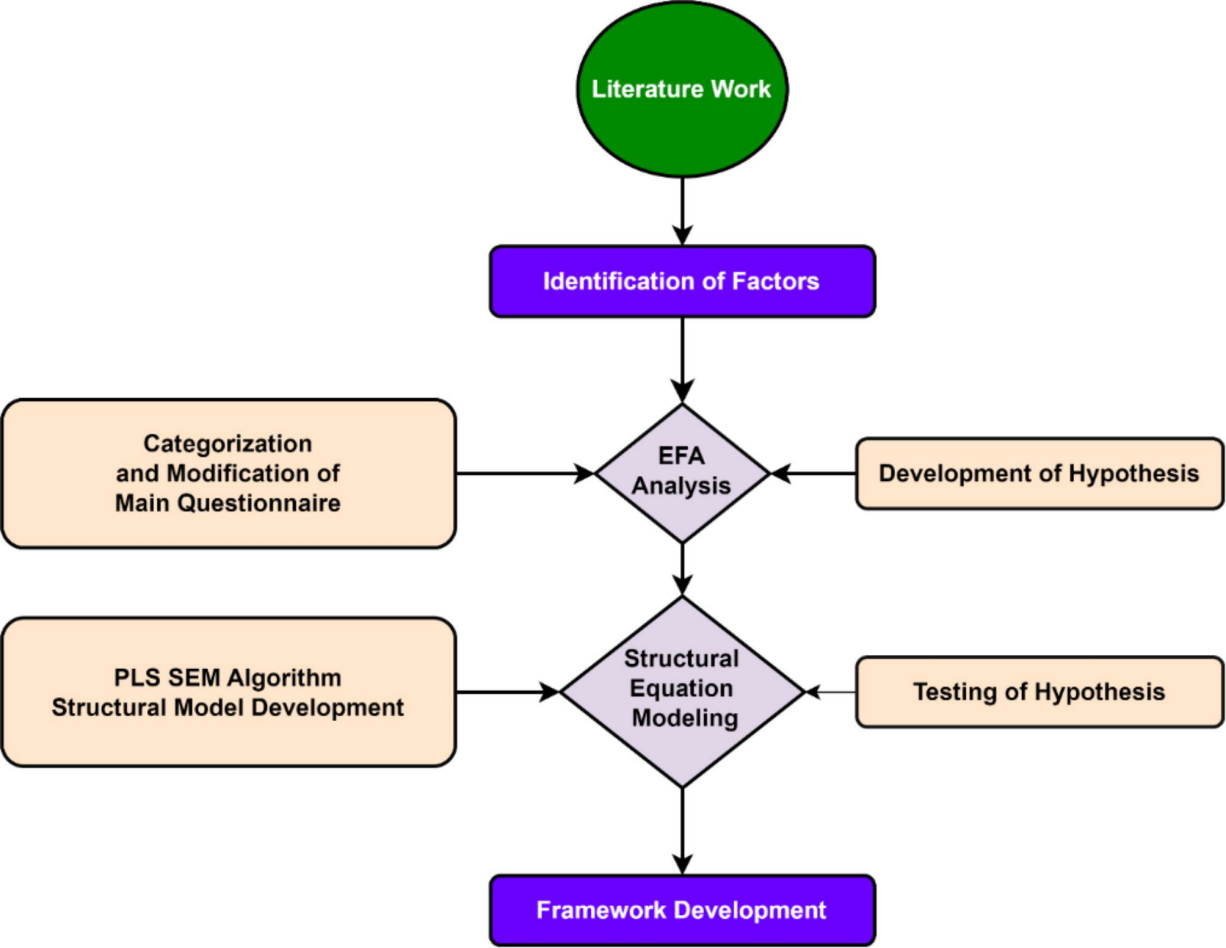
Flow chart of the study.

This study involved three major phases. In the first phase, an in-depth literature review was conducted to identify the factors indicating the application of IoT for the safety and efficiency of construction sites to make them smart sites^29^. In the second phase, the quantitative analysis and the hypothesis are developed. In the last stage, phase depth, SEM analysis is carried out to verify the hypothesis of the studies. The participants in this study were construction professionals working in various roles on site, including project managers, site engineers, safety officers, and labor supervisors. Field professionals who distributed the survey included experienced site supervisors and project managers who were trained to ensure impartiality during the survey process. To mitigate any potential bias, surveys were administered in a confidential manner, ensuring that respondents felt no pressure or influence from their supervisors. The anonymity of responses was emphasized to the participants to reduce the likelihood of response bias. Figure 1 shows the complete theme of the study. Partial Least Squares Structural Equation Modeling (PLS-SEM) was chosen due to its suitability for exploratory research and its ability to handle complex models with small to medium sample sizes effectively. While our size is adequate, PLS-SEM provides robustness against non-normal data and is preferred for its predictive accuracy and flexibility in handling formative constructs, which are key aspects of this study. The questions in the questionnaire were developed based on an extensive literature review and consultation with industry experts. Key factors and indicators relevant to IoT implementation in construction were identified from prior studies, and the questions were designed to capture these variables effectively. The questionnaire was pre-tested with a small group of professionals to ensure clarity and relevance.

### Identification of factors

**Fig. 2:**
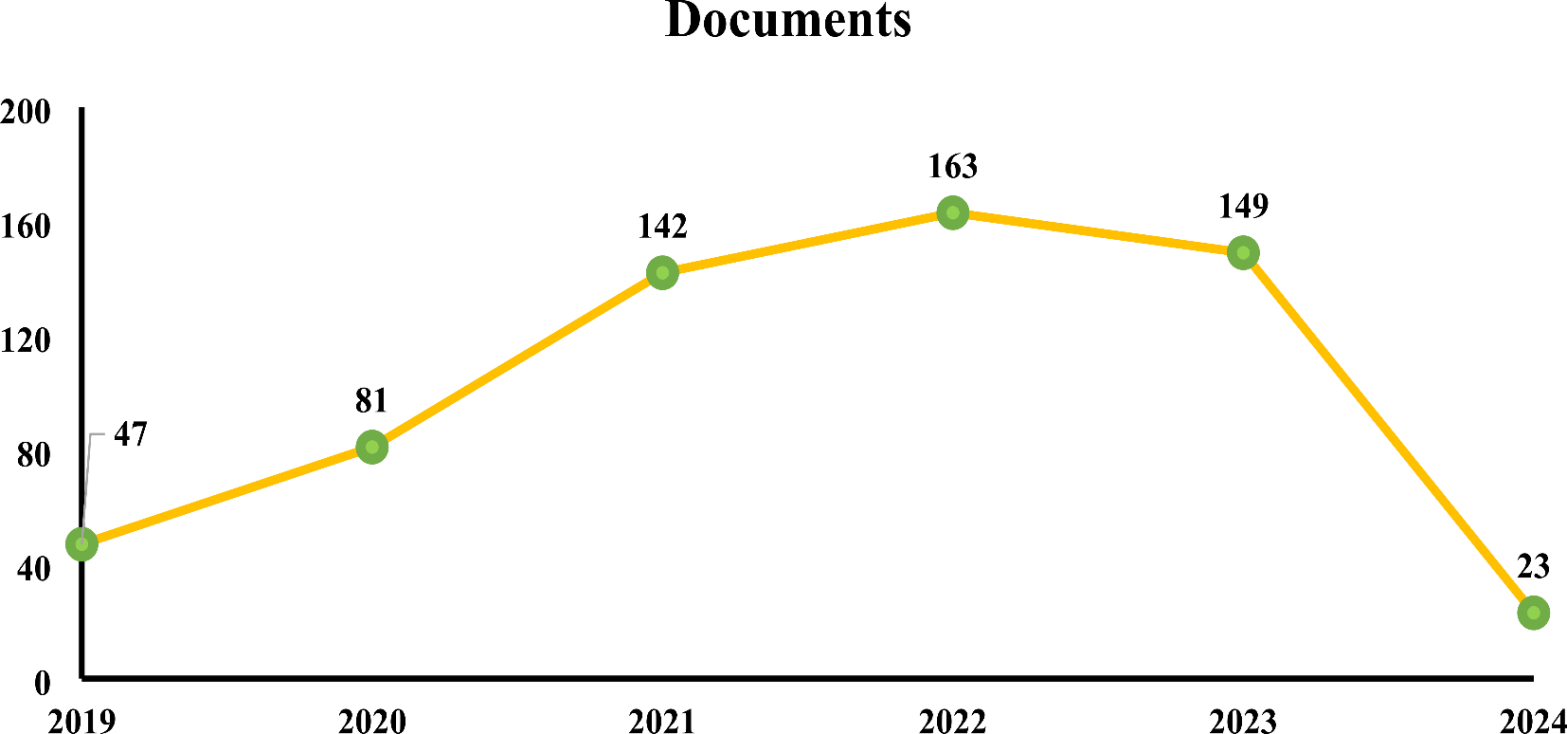
Documents from 2019 onwards.

A systematic literature review was conducted to identify key factors for Internet of Things (IoT) implementation on construction sites. The Scopus database was searched using the keywords “IoT” AND “Construction” AND “Sites” OR “Implementation” OR “Safety” OR “Efficiency” in article titles, abstracts, or keywords. This broad search returned 1,618 documents initially. The findings were filtered to only contain English-language documents from 2019 through February 2023 that were categorized as technical papers or review articles in Scopus, in order to concentrate on recent, high-quality publications. This narrowed the results down to 605 relevant documents. Analysis of publication dates showed steady growth in research on this topic. In 2022, 163 papers were published, up from 149 in 2023 and 142 in 2021 as shown in Fig. 2.

**Fig. 3:**
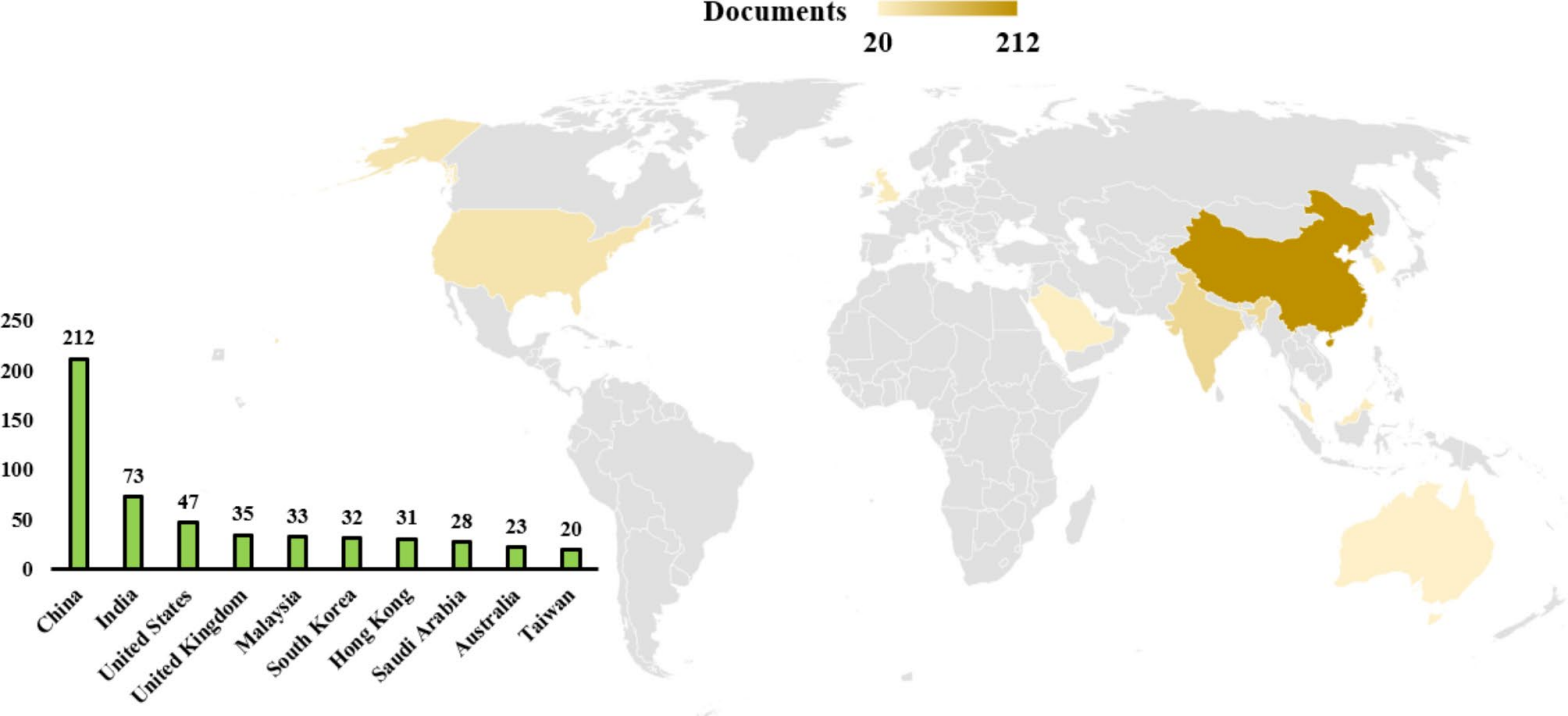
Documents per Country from 2019 onwards.

The most publications originated from China (212), India (73), United States (47) and United Kingdom (35), indicating significant research interest in these countries as shown in Fig. 3. VOS viewer software was used to visualize bibliometric networks in order to identify technological application areas and analyze keyword usage. Clusters centered on important subjects such wireless sensors, asset tracking, safety, augmented reality, sustainability, and construction productivity were identified by keyword co-occurrence analysis.

**Fig. 4:**
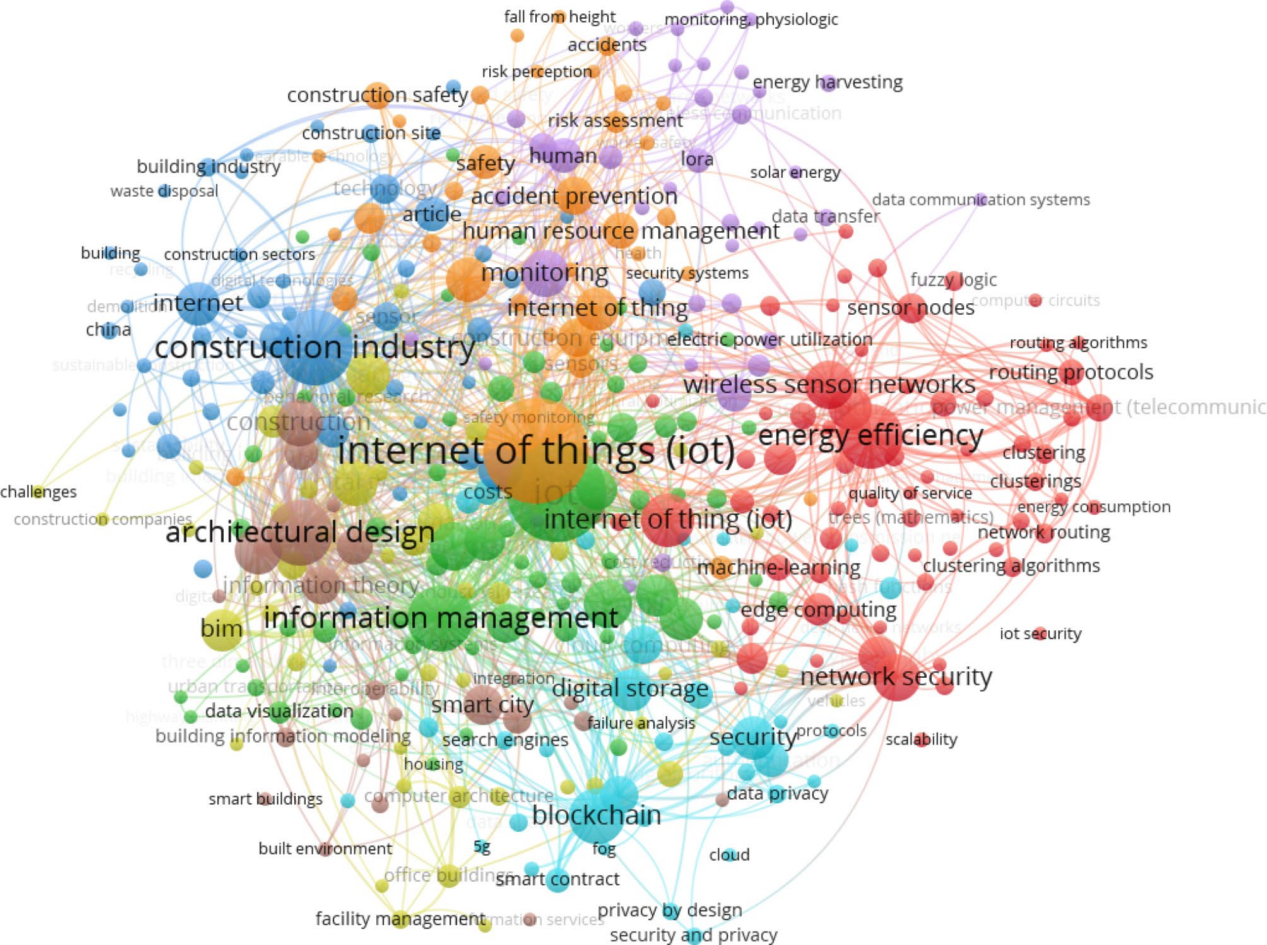
VOS visualization of keywords.

This provided insights into research domains and guided factor selection. Full text review of the filtered literature corroborated the key factors derived from the visualized keyword analysis. The most prevalent factor categories were worker-centered technologies like wearables, handhelds, and exoskeletons to improve safety and productivity. Location tracking of workers, vehicles and equipment was another key focus area in the papers. Environmental monitoring, structural health monitoring, materials management, and data analytics also featured prominently as shown in Fig. 4.

The structured methodology enabled effective identification of a large set of candidate documents for review. Filtration criteria removed low-quality publications and narrowed the focus on construction sites. The bibliometric analysis provided visual guidance to focus the review on papers discussing key factors aligned with major application categories for IoT on sites. This rigorous, transparent, and replicable approach demonstrates systematic literature analysis to derive factors from evidence in the scholarly domain.

The implementation of IoT technology on construction sites could potentially yield several benefits, including enhanced employee welfare, increased adherence to safety regulations, improved renovation scheduling, enhanced device functionality, and ultimately more streamlined operations. The Internet of Things has enabled access to vast quantities of data from construction sites. Heavy equipment that was formerly quiet is now able to open in real time, enabling predictive maintenance plans to prevent issues before they arise. Ensuring worker wellness and regulatory compliance, data is also changing the environment. Wearable technology even makes the human body a source of valuable data, enabling personalized work schedules and tiredness tracking^13,14^. Data analysis yields insights that improve resource allocation and construction processes^15,16^. Table 2 also indicates that implementing IoT systems facilitates the administration of energy consumption and enhances operational efficiency. Employee monitoring in real-time ensures safety and expedites emergency response. Integration of IoT into construction sites results in enhanced safety, productivity, and cost-effectiveness.

**Table 2:** Identified factors for IoT implementation for the safety and efficiency of smart construction sites.

Variables	Description	References
IOT-SE01	Sensors of the Internet of Things collect data in real-time on equipment performance, usage patterns, and operating conditions.	^17,18^
IOT-SE02	Optimal maintenance planning increases equipment dependability and longevity.	^1,2^
IOT-SE03	Early defect or malfunction detection enables proactive maintenance to minimize downtime.	^3,4^
IOT-SE04	Real-time surveillance ensures compliance with safety regulations and promotes a healthier workplace.	^5,19^
IOT-SE05	Predictive maintenance models facilitate proactive maintenance scheduling, thereby minimizing unscheduled delays.	^7,8^
IOT-SE06	Data on fuel consumption, engine heath, and operating conditions facilitates equipment utilization optimization.	^6^
IOT-SE07	IoT-based wearable devices can detect falls or incidents and automatically activate distress signals for prompt assistance.	^9,10^
IOT-SE08	Environmental monitoring improves site-wide security, worker health, and sustainability.	^20,21^
IOT-SE09	In high-risk areas, Internet of Things sensors can detect hazardous conditions and alert workers and managers to prevent accidents.	^22,23^
IOT-SE10	Tracking the location of employees in real-time ensures their safety and enables effective emergency response.	^24,25^
IOT-SE11	Internet of Things sensors measure environmental parameters such as air quality, noise levels, temperature, and humidity.	^17,26^
IOT-SE12	Wearable devices facilitated by the Internet of Things, such as smart headgear and safety garments, monitor employees’ vital signs and fatigue levels in real time.	^18,27^
IOT-SE13	Enhanced equipment management increases productivity and reduces expenses.	^28,29^
IOT-SE14	Insights derived from data improve resource allocation and optimize construction processes.	^27,28^
IOT-SE15	IoT systems allow for the management and optimization of energy consumption.	^18,29^
IOT-SE16	In construction locations, predictive analytics improve operational efficiency and productivity.	^14,30^

### Data collection

Pakistan’s Mirpur region was the focus of a quantitative investigation into the impact of IoT implementation on construction sites. Multiple studies have shown that sample sizes of around 100 can yield reliable results when the sample is representative, and the data is well-analyzed. For instance, studies by Westland^99^ demonstrate that sample sizes in the range of 100–150 are sufficient for SEM when the data quality is high, and the model is not overly complex^100^. These studies are applicable to our case as our SEM model is straightforward and the data collected is of high quality. The 188 survey respondents provided 114 valid responses, which were considered for data analysis. Field professionals sometimes delivered the survey by hand in addition to via email^3,4^. The response rate for the current research was around 60.6%. The survey employed a Likert scale consisting of five points to assess the degree to which respondents concurred or disapproved of specific record-accumulating areas. This scale provided a well-established framework for evaluating the perspectives and opinions of participants regarding the benefits of deploying IoT on production websites. Multiple studies in analogous contexts have demonstrated that effects originating from sample sizes as small as one hundred can still be genuine and precise. It is determined that a sample size of 114 respondents is adequate for the purposes of this investigation. Furthermore, by focusing on the Mirpur region, the statistics become more precise and relevant, thereby enhancing our understanding of the implementation of IoT in the construction sector.

### Exploratory factor analysis (EFA) analysis for hypothesis development

The variables identified in the literature were classified into four groups using an exploratory factor analysis (EFA), and hypotheses were developed for this analysis. The EFA analysis made it possible to identify latent patterns and additives within the data and classify the variables according to the characteristics they shared. The EFA evaluation has effectively categorized variables into distinct classes, providing a systematic framework for developing research inquiries and hypotheses. This approach facilitated the elucidation of the interconnections between the variables and provided direction for the subsequent assessment and interpretation of the study’s findings. For EFA evaluation, the subsequent equation is applied.

i $$X=\lambda LF + E$$

Where X: variables or signs that were noticed, λ: Factor loadings: the strength of the connection between latent factors and observable variables, Latent variables (basic ideas), E: The variance or error connected to every variable that was observed. The formula shows how the latent factors (LF) affect the observable variables (X) via factor loadings (FL), with the error term (E) taking care of any variation that cannot be explained.

### PLS-SEM measurement model

The relationships between the variables of the study were analyzed using Partial Least Squares Structural Equation Modelling (PLS-SEM), which was implemented using the Smart PLS 4 software. Robust statistical methods, such as PLS-SEM, are optimal for examining intricate relationships with limited sample sizes while also facilitating comparisons of model sizes and structures. The investigation encompassed the assessment of both discriminant and convergent validity. The degree to which the signs (observed variables) of each latent construct (underlying element) converge and measure the same concept is convergent validity. In order to reach this conclusion, an analysis was conducted on the composite reliability (CR), average variance extracted (AVE), and indicator loadings for each construct. Determining exceptional convergent validity required indicator loadings to surpass the predetermined threshold, AVE values to exceed 0.5, and CR values to surpass 0.70.

### Structural model analysis

Bootstrap resampling techniques were employed to examine the four research hypotheses during the structural model analysis. Determining the significance and direction of the relationships between the latent variables in the study model became the objective of this investigation. Utilizing the bootstrap system, the sample mean (M), standard deviation (STDEV), t-stats, p-values, and original sample (O) could be calculated. The bootstrap method generated a distribution of the path coefficients for each hypothesis through the iterative resampling of the statistics. Using this distribution, the actual sample coefficients (O) have been computed in order to determine the effect size. The calculation of the sample mean (M) and standard deviation (STDEV) was performed to assess the coefficients’ dispersion and significance. Q2 is a statistical metric employed in Partial Least Squares Structural Equation Modelling (PLS-SEM) to evaluate the capability of a research model to make predictions. The metric assesses the accuracy with which the model forecasts outcomes based on latent constructs, unbiased elements, or structured variables. The investigation of the four research hypotheses was conducted using bootstrap resampling techniques.

## Results and analysis

### Demographic details

Table 3 provides specifics on the categories, classifications, frequencies, and percentages of the respondents. According to profession, the respondents included quantity surveyors (6.9%), architects (9.1%), civil engineers (23.4%), M&E engineers (5.1%), project managers (14.3%), and others (6.9%). In terms of organization, the respondents represented Contractors (25.1%), Consultants (21.7%), and Clients (18.2%). In the construction business, 23.4% of respondents had no experience, 18.9% had, 11.4% had 11–15 years, 4.6% had 16–20 years, and 6.8% had more than 20 years. 61.7% of respondents said they were knowledgeable about digital technologies (IoT), while 3.4% said they weren’t/may not be. The table offers a thorough analysis of respondent characteristics, which will facilitate understanding of the study’s conclusions and ramifications.

**Table 3:** Demographic details of respondents.

Category	Classification	Frequency	%
Profession	Quantity Surveyor	12	6.9
	Architect	16	9.1
	Civil Engineer	41	23.4
	M&E Engineer	9	5.1
	Project Manager	25	14.3
	Other	11	6.3
Organization	Contractor	44	25.1
	Consultant	38	21.7
	Client	32	18.3
Construction Industry Experience	0–5 Years	41	23.4
	6–10 Years	33	18.9
	11–15 Years	20	11.4
	16–20 Years	8	4.6
	Over 20 Years	12	6.9
Knowledge of Digital Technologies (IoT)	Yes	108	61.7
	No/Maybe	6	3.4

### EFA analysis

The EFA analysis was conducted to investigate the variables’ underlying factors, as shown in Table 4. The analysis revealed that the eigenvalues for the components were greater than 1, indicating that these components account for a considerable portion of the data’s variance^24,26^. Loading values greater than 0.60 were deemed significant, indicating a robust relationship between the variables and their respective components^27,28^.

The variables were labelled IOT-SE01, IOT-SE02, IOT-SE03, etc., to represent specific items related to the construction industry IoT focus of the study. Variables with loading values below 0.60 were excluded from the analysis because they lacked a significant association with any component^15,16^. Principal Component Analysis was used for data extraction, which helps identify the underlying factors that explain the variance in the data^14,18^. Varimax with Kaiser Normalization was used as the rotation method, which aids in attaining simplified and more interpretable factor structures^3,7^. The EFA analysis revealed the presence of significant components and their respective variables while excluding one factor. Cronbach’s alpha values evaluated the internal consistency within each component, bolstering the analysis’s credibility.

**Table 4:** Exploratory factor analysis output.

Variables	Component				Cronbach Alpha
	1	2	3	4	
IOT-SE12	0.869				0.819
IOT-SE09	0.824				
IOT-SE07	0.668				
IOT-SE10	0.609				
IOT-SE13		0.822			0.795
IOT-SE06		0.789			
IOT-SE03		0.664			
IOT-SE08			0.794		0.711
IOT-SE15			0.761		
IOT-SE04			0.699		
IOT-SE11			0.666		
IOT-SE01				0.765	739
IOT-SE05				0.709	
IOT-SE14				0.645	

“Extraction Method: Principal Component Analysis.”

“Rotation Method: Varimax with Kaiser Normalization.”

“Variable IOT-SE02, IOT-SE16 excluded because of loading less than 0.6.”

**Fig. 5:**
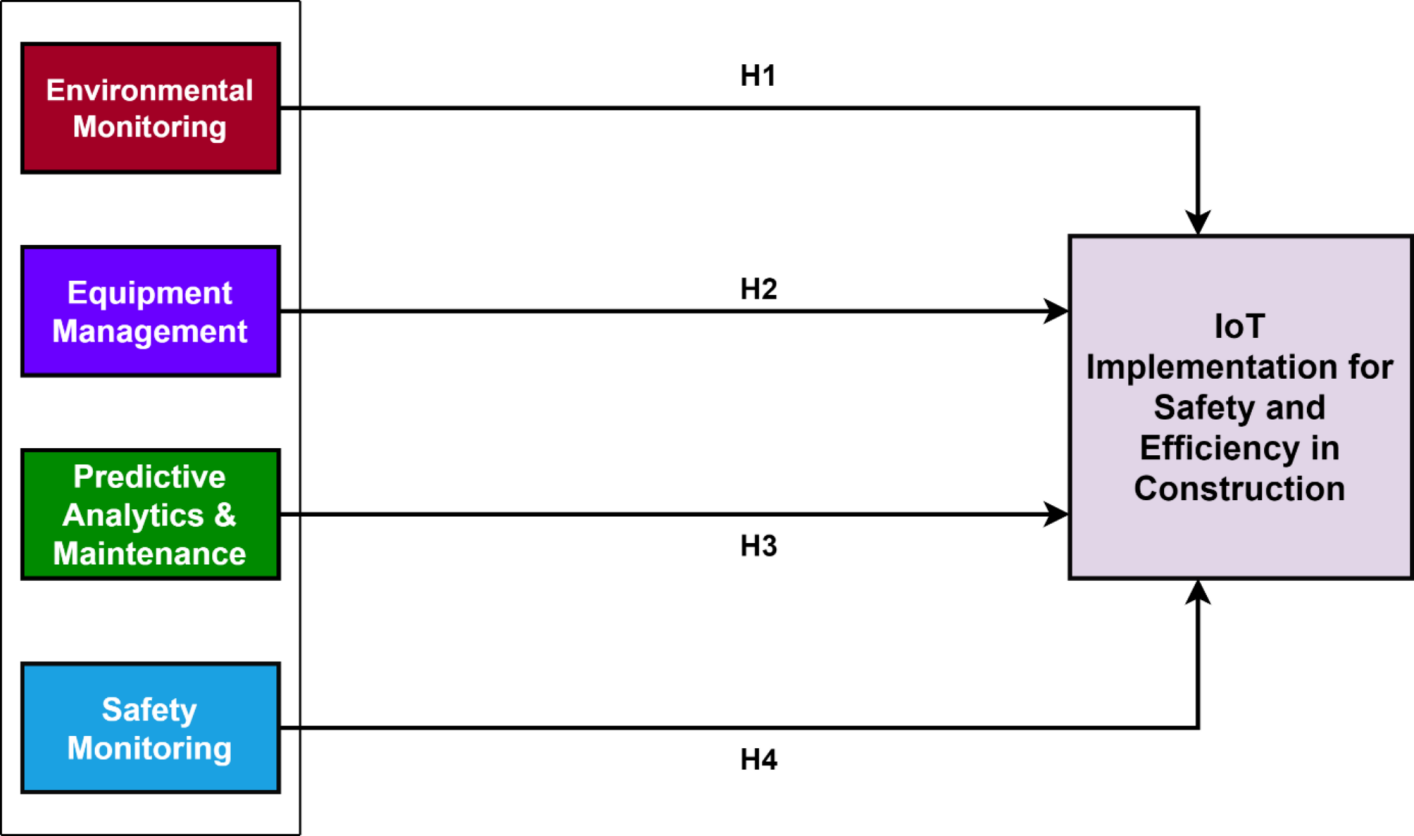
Hypothesis of the study developed after EFA analysis.

Based on the results of the EFA and the classification of variables into four groups, four hypotheses concerning the significant relationships between these groups and the implementation of IoT on construction sites were formulated, which are described in Fig. 5.

- H1: The relationship between environmental monitoring and the implementation of IoT on construction sites is substantial.
- H2: The relationship between equipment management and the implementation of IoT on construction sites is substantial.
- H3: There is a significant relationship between predictive analytics and maintenance and the implementation of IoT on construction sites.
- H4: There is a significant relationship between safety monitoring and the implementation of IoT on construction sites.

### PLS-SEM model development

Table 5 demonstrates the consistency and intensity of the relationships between the observed variables and their respective constructs, as determined by the convergent validity analysis. The variable IOT-SE12 has a high loading of 0.784 for the Safety Monitoring construct, indicating a significant relationship with this construct. This indicates that IOT-SE12 measures the intended construct effectively^9,10^. The VIF value 1.237 indicates minimal multicollinearity, indicating that the observed variables within this construct are distinct. The Safety Monitoring construct’s internal consistency is supported by a Cronbach Alpha (CA) value of 0.777, indicating high reliability^5,22^. It is further supported by the Composite Reliability (CR) score of 0.779% that the observed variables in this construct are reliable. The Safety Monitoring construct captures a significant amount of variation in the observed variables, as shown by the Average variation Extracted (AVE) value of 0.54, showing sufficient convergent validity^6,21^.

**Table 5:** Convergent validity results.

Cluster	Assigned code	Loadings	VIF	CA	CR	AVE
Safety Monitoring	IOT-SE12	0.784	1.237	0.778	0.779	0.54
	IOT-SE09	0.716	1.213			
	IOT-SE07	Deleted	1.239			
	IOT-SE10	0.703	1.157			
Equipment Management	IOT-SE13	0.865	2.038	0.775	0.867	0.685
	IOT-SE06	0.746	1.964			
	IOT-SE03	0.867	2.854			
Environmental Monitoring	IOT-SE08	0.797	2.141	0.741	0.803	0.578
	IOT-SE15	0.693	2.328			
	IOT-SE04	0.786	2.312			
	IOT-SE11	Deleted	1.223			
Predictive Maintenance and Analytics	IOT-SE01	0.795	1.224	0.882	0.929	0.814
	IOT-SE05	0.952				
	IOT-SE14	0.950				

CA = Cronbach Alpha; CR = Composite Reliability; AVE = Average Variance Extracted

The findings of this study indicate that the variables comprising the Safety Monitoring construct consistently recreate consistent relationships and precisely measure the intended construct, thereby contributing to the convergent validity of the assemblages. The absence of HTMT ratios for the Environmental Monitoring construct in Table 6 HTMT ratios hinders the ability to discern how it differs from the other constructs. The fact that environmental tracking is discernible and separate from the other components of the study suggests that it possesses exceptional discriminant validity. A few overlaps between the opposite additives, Safety Monitoring (SMO), Predictive Analytics & Maintenance (PAM), and Equipment Management (EMA), are indicated by the HTMT ratios. It is crucial to remember that the HTMT ratios provide sufficient discriminant validity among those concepts, even if they fall below the critical 0.85 threshold.

**Table 6:** Results of HTMT discriminant validity.

Constructs	EMO	EMA	PAM	SMO
Environmental Monitoring				
Equipment Management	0.548			
Predictive Analytics & Maintenance	0.325	0.4		
Safety Monitoring	0.082	0.635	0.242	

Even though there is some overlap between the constructs, the HTMT ratios indicate that the constructs in your study have adequate discriminant validity. This indicates that the constructs are sufficiently distinct to be regarded as separate and independent, supporting the study’s measurement model’s reliability^4,19^.

The Fornell-Larcker criterion results in Table 7 support the discriminant validity of the measurement model’s constructs. The square root of the average variance extracted (AVE) for each construct is greater than the correlations with other constructs, indicating that the constructs are distinctive. Environmental Monitoring (EMO) demonstrates discriminant validity with an AVE square root 0.76, exceeding correlations with other constructs. This demonstrates that EMO differs from the other variables^6,8^. Equipment Management (EMA) partially satisfies the criterion because it has a higher correlation with Predictive Analytics & Maintenance (PAM) than with Safety Monitoring (SMO). However, additional analysis is required to establish the full validity of discrimination between EMA and SMO. With a square root of AVE of 0.902, Predictive Analytics & Maintenance (PAM) demonstrates discriminant validity, superseding correlations with other constructs. This demonstrates that PAM differs from the other variables^9,21^.

**Table 7:** Fornell lacker criterion for discriminant validity.

Constructs	EMO	EMA	PAM	SMO
Environmental Monitoring = EMO	0.76			
Equipment Management = EMA	0.469	0.828		
Predictive Analytics & Maintenance = PAM	0.255	0.332	0.902	
Safety Monitoring = SMO	0.681	0.466	0.172	0.735

Table 8 indicating the cross-loading criterion was used to evaluate the discriminant validity of the measurement model’s variables. It investigates the correlations between each variable and its respective construct and the correlations between the variable and other constructs^6,19^. The variables are evaluated in the supplied table based on their cross-loadings with the constructs. To establish discriminant validity, a variable’s cross-loadings with its construct should be greater than those with other constructs^5,8^. The results indicate that most variables have higher cross-loadings with their respective constructs, providing evidence for discriminant validity. For instance, IOT-SE08 has a greater cross-loading with Environmental Monitoring (EMO) than other constructs. Similarly, IOT-SE13 has a greater cross-loading with Equipment Management (EMA), Predictive Analytics & Maintenance (PAM), and Safety Monitoring (SMO) than IOT-SE01 and IOT-SE05.

**Table 8:** Cross-loading criterion for discriminant validity.

Variables	EMO	EMA	PAM	SMO
IOT-SE08	0.797	0.583	0.252	0.579
IOT-SE15	0.693	0.198	0.193	0.41
IOT-SE04	0.786	0.21	0.123	0.543
IOT-SE13	0.212	0.865	0.274	0.301
IOT-SE06	0.638	0.746	0.266	0.528
IOT-SE03	0.198	0.867	0.275	0.252
IOT-SE01	0.147	0.324	0.795	0.119
IOT-SE14	0.262	0.294	0.95	0.161
IOT-SE05	0.268	0.288	0.952	0.181
IOT-SE09	0.406	0.27	0.015	0.716
IOT-SE10	0.589	0.447	0.179	0.703
IOT-SE12	0.479	0.286	0.163	0.784

EMO = Environmental Monitoring; EMA = Equipment Management; PAM = Predictive Analytics & Maintenance; SMO = Safety Monitoring

Most variables demonstrate discriminant validity based on the cross-loading criterion, as they exhibit higher cross-loadings with their respective constructs than other constructs.

### Structural model development

A bootstrap analysis was conducted to assess the robustness and dependability of the study’s hypotheses, as shown in Table 9. To generate bootstrap samples, it was necessary to resample the data and calculate statistics for each sample repeatedly. The original sample (O), sample mean (M), standard deviation (STDEV), t-statistics, and p-values are provided to summarize the results of the bootstrap analysis^21,22^. These statistics aid in assessing the significance of the interrelationships between variables. The bootstrap analysis indicates that all hypotheses have reached statistical significance. The relatively large t-statistics for each hypothesis indicate a robust relationship between the independent and dependent variables^6,8^. Each hypothesis is reported with a p-value of 0, indicating a statistically significant relationship.

**Table 9:** Hypothesis of the study.

Hypothesis	Relation	(O)	(M)	(STDEV)	T statistics	*P* values	Results	Q^2^ (= 1- SSO/SSE)
H1	EMO -> IoT	0.38	0.379	0.014	27.405	0	Accepted	–
H2	EMA -> IoT	0.343	0.342	0.011	32.318	0	Accepted	–
H3	PAM -> IoT	0.222	0.219	0.029	7.691	0	Accepted	–
H4	SMO -> IoT	0.369	0.369	0.014	25.848	0	Accepted	–
–	IoT IMP-SE	–	–	–	–	–	–	0.169

(O) = Original sample; (M) = Sample mean; (STDEV) = Standard deviation; EMO = Environmental Monitoring; EMA = Equipment Management; PAM = Predictive Analytics & Maintenance; SMO = Safety Monitoring; IoT IMP-SE = IoT Implementation for Safety and Efficiency; Q^2^ = Predictive Relevance.

These results indicate that the study’s findings are reliable and consistent, as multiple bootstrap samples support them. The bootstrap analysis supports the hypotheses and strengthens the validity of the study’s conclusions. The predictive relevance analysis indicates that the model’s predictive power is moderate. The sum of squares explained (SSO) is 9056.000, indicating that the model accounts for this variation in IoT implementation for safety and efficiency. Table 10 shows the GOF indices for the structural model in addition to the Structural Model Output Standardized Estimates Path Diagram. These indices evaluate the model’s overall fit to the data, ensuring an accurate evaluation of the model’s applicability. The GFI, RMSEA, and Chisq Absolute Fit Indices, among others, all came within acceptable bounds, suggesting a satisfactory fit between the model and the data. The Incremental Fit Indices, such as the TLI and the CFI, which are both above the recommended values, also showed an appropriate fit with respect to the baseline model. The Parsimonious Fit Index, or Chi-square/Degrees of Freedom, or Chisq/df ratio, eventually fell below the recommended cutoff, indicating a modest and well-fitting model.

**Table 10:** Goodness of fit (GoF) measures.

Category	Index Name	Index	Attained Values	Acceptance criteria
Absolute Fit Indices	Discrepancy Chi square	Chisq	579.91	*p* > 0.01
	Goodness of Fit Index	GFI	0.931	> 0.90
	Root Mean Square of Error	RMSEA	0.064	< 0.08
Incremental Fit	Comparative Fit Index	CFI	0.925	> 0.90
Indices	Tucker-Lewis Index	TLI	0.912	> 0.90
Parsimonious Fit	Chi Square/Degree of freedom	Chisq/df	1.801	< 3

**Fig. 6:**
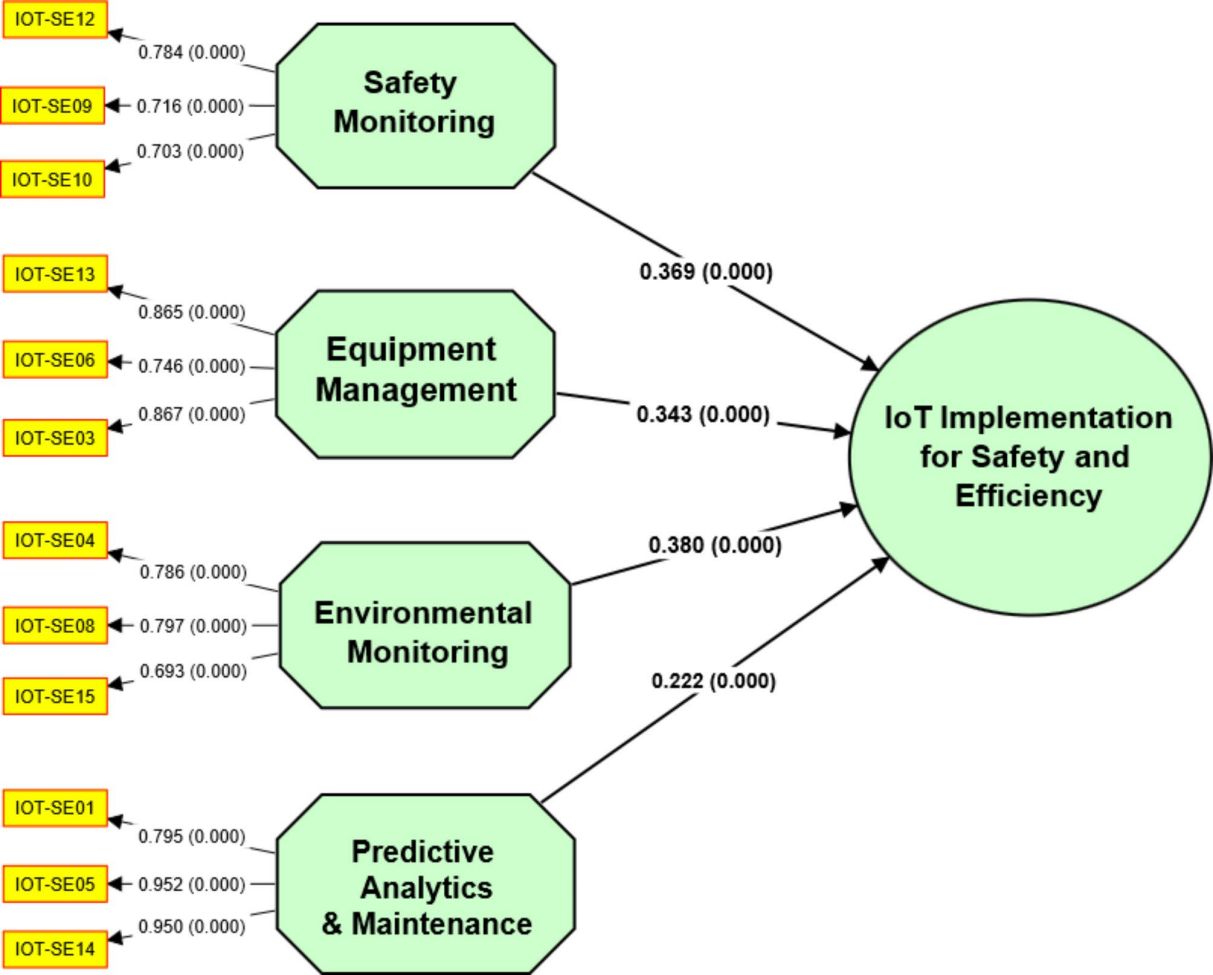
Path loadings with p-values.

Figure 6 depicts the path loadings and associated statistical results for each of the study’s hypotheses. It demonstrates the intensity of the relationship between the independent variable the dependent variable (IoT implementation) and pertinent statistical measures. The path loadings are the coefficients that have been normalized to indicate the magnitude and direction of the relationship between the variables. These indices represent the influence of each independent variable on the dependent variable.

**Fig. 7:**
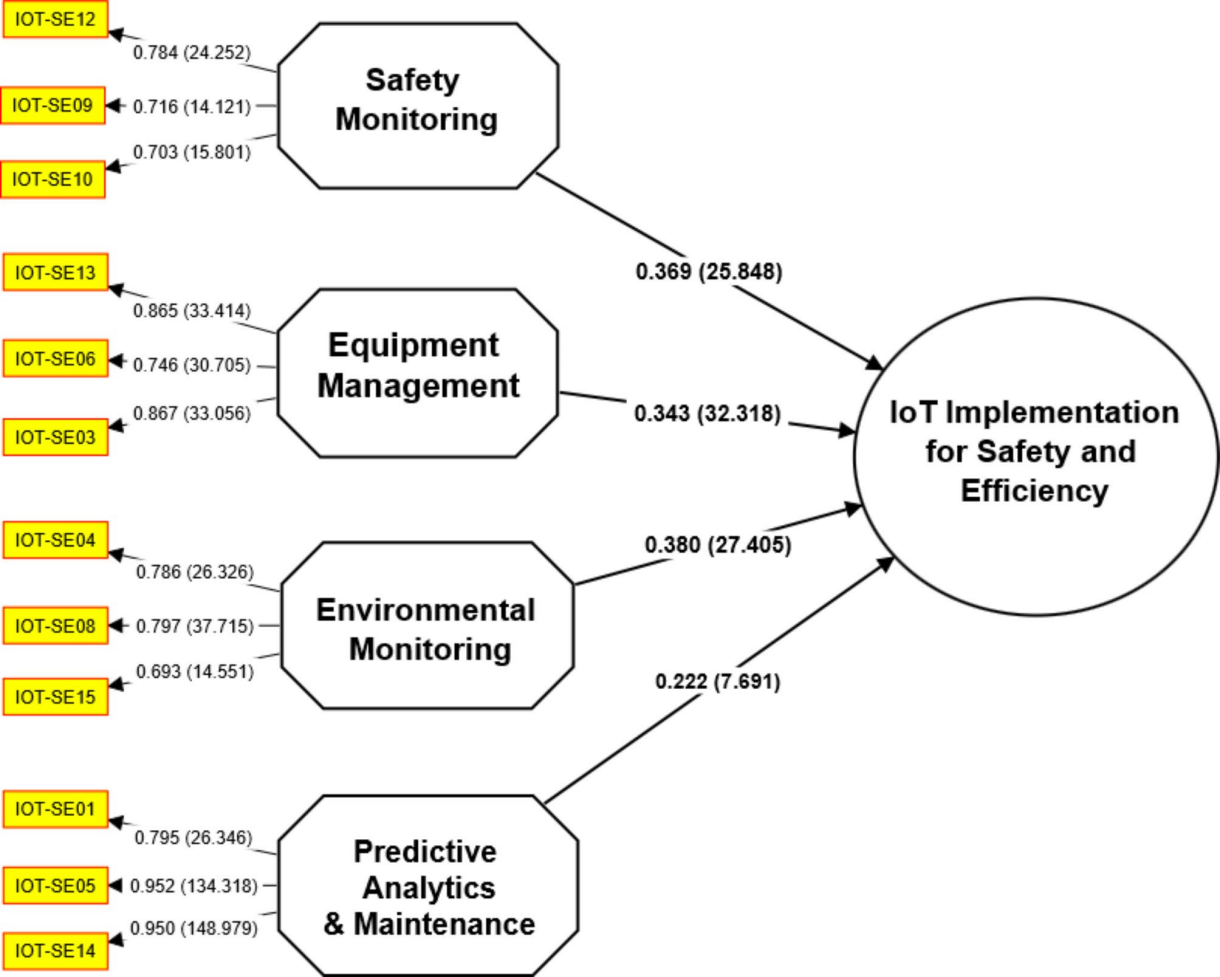
Path Loadings with T stats values.

Figure 7 illustrates the T-statistics for each hypothesis, which evaluate the significance of the relationship between the independent variables (EMO, EMA, PAM, and SMO) and the dependent variable (IoT implementation). T-statistics quantify the intensity of the relationship between variables, considering sample size and variance^7,19^. Higher T-statistics indicate a more significant and robust relationship. In this instance, the T-statistics for all four hypotheses (H1, H2, H3, and H4) are relatively substantial, indicating a robust and statistically significant relationship between the independent variables and IoT implementation.

Based on the T-statistics, we can conclude that the data validate the hypothesized relationships and that the independent variables significantly affect IoT implementation.

## Discussion

**Fig. 8:**
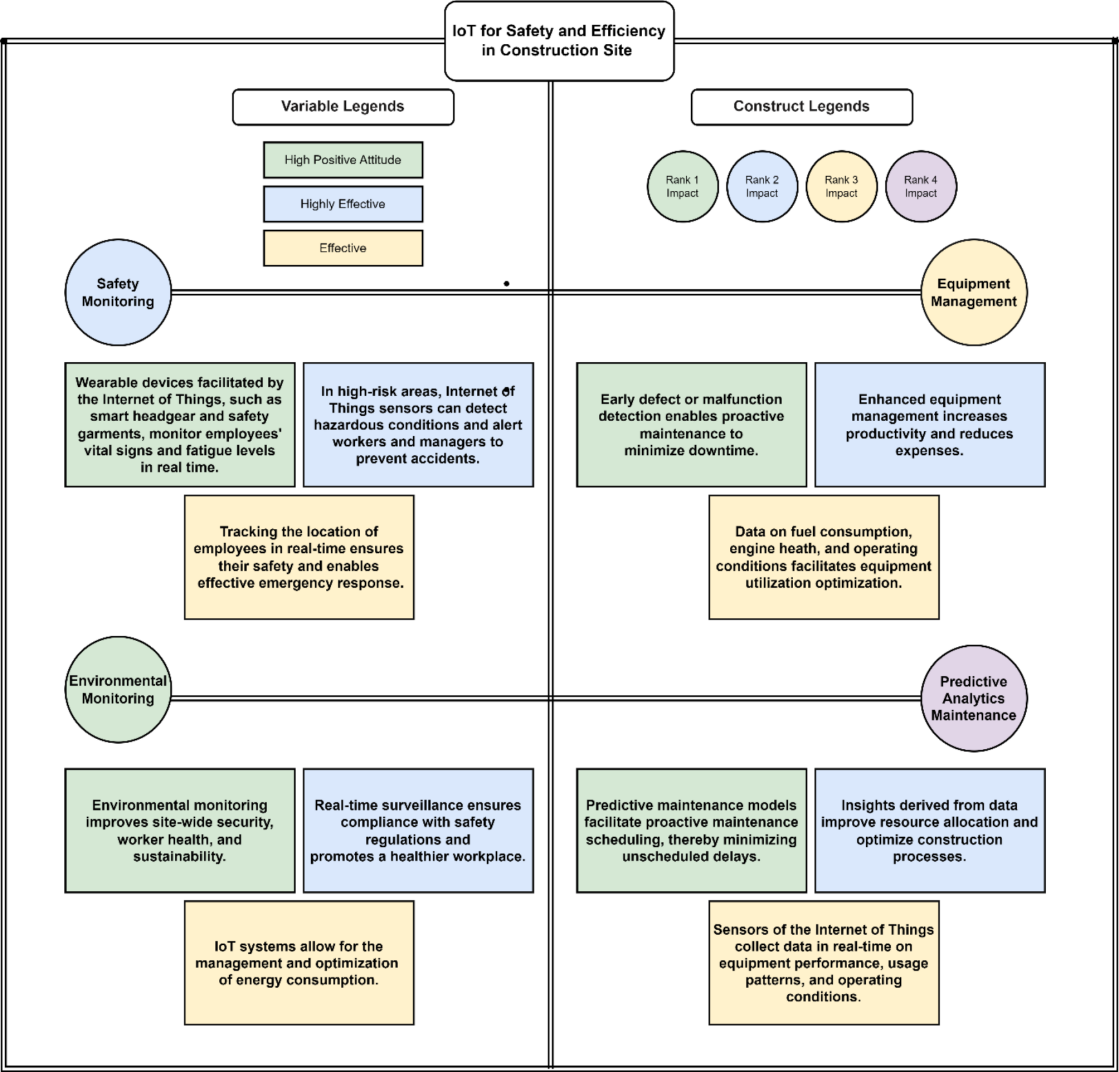
Framework for IoT considering safety and efficiency of construction operations.

The study examined four hypotheses regarding the relationships between various variables and the implementation of IoT for construction site safety and efficiency. Significant results supported all of the hypotheses. Focusing on the Mirpur region allows for a detailed and context-specific analysis of IoT implementation in construction, which enhances the precision and relevance of the data. The region’s active construction industry and unique challenges provide a rich dataset that reflects real-world conditions, making the findings highly applicable and actionable for similar developing regions. Figure 8 is framework is explaining the all the hypothesis with variables considering their effectiveness in terms of IoT for safety and efficiency in construction site operations.

The path coefficient for Hypothesis 1 (H1), which suggests a relationship between Environmental Monitoring (EMO) and IoT implementation, was 0.38. The original sample was analyzed, and the mean and standard deviation values were calculated accordingly. The T statistic value was 27.405, indicating a highly significant relationship^8,10^. The validity of Hypothesis 1 was supported by the finding that the P value was 0. Hypothesis 2 (H2)’s route coefficient, which postulated a connection between IoT adoption and Equipment Management (EMA), was 0.343. The statistical study, which included the mean, standard deviation, T statistic value of 32,318 and P value of 0, verified the significance of the link. The third hypothesis (H3) focused on the connection between IoT installation and Predictive Analytics & Maintenance (PAM). The positive correlation within reason is supported by the path coefficient of 0.222. Hypothesis H3 was substantiated by the T statistic of 7.69 and the P value less than 0.005. The fourth and ultimate hypothesis (H4) assessed the relationship between the deployment of IoT and Safety Monitoring (SMO). A significant and favorable correlation was identified through the utilization of the path coefficient of 0.369. The T statistic value of 25.848 and the P statistic value of 0 provided persuasive support for adopting H4. These facts illustrate the importance of every aspect pertaining to the implementation of the IoT regarding the overall efficiency and integrity of construction work sites. By analyzing critical connections, the significance of Environmental Monitoring, Equipment Management, Predictive Analytics & Maintenance, and Safety Monitoring to the successful adoption and utilization of the IoT era by the construction company is determined.

## Conclusion

The objective of this investigation was to examine the correlations between various factors and the effectiveness and safety of construction sites utilizing the IoT. The results of the study, which demonstrated a strong correlation between the attributes and IoT adoption, provided support for all four hypotheses. Integrating IoT within a construction zone necessitates an emphasis on environmental monitoring, machine and equipment administration, predictive analytics and protection, and safety monitoring, as demonstrated by the results. The robust route loadings and statistical significance of T records indicate the strength of these interactions. Beneficiaries of these findings include construction professionals, job administrators, and safety stakeholders due to their practical implications. The study emphasizes the criticality of incorporating IoT generation into construction strategies to enhance operational efficiency and safety. Construction sites have the potential to leverage the IoT to enhance efficiency and mitigate risks through environmental monitoring, device management, predictive analytics-driven maintenance, and safety assurance. The studies contribute to the body of knowledge by substantiating the significance of those variables in the context of construction industry. It provides policymakers and practitioners with crucial insights regarding the critical factors that influence the effective implementation of the IoT era. Nonetheless, it is critical to acknowledge the limitations of the examination. The investigation employed a restricted geographic area and a modest sample size. To enhance the reliability of the findings, future research may wish to expand the scope of interest to include more geographical regions and increase the scale of the pattern. Additionally, qualitative research methods can be utilized to gain a more comprehensive understanding of the perspectives and experiences of construction professionals regarding the incorporation of IoT. This examination concludes by emphasizing the significance of environmental monitoring, equipment management, predictive analytics and security, and protection monitoring when utilizing IoT for construction and development efficiency and security. The results enhance the existing body of knowledge and provide practical suggestions for professionals in the field, thereby promoting the environmentally friendly incorporation of IoT technology within the construction site.

## Electronic supplementary material

Below is the link to the electronic supplementary material.

Supplementary Material 1

## Data Availability

All data generated or analysed during this study are included in this published article [and its supplementary information files].
