# Supplementary material for: Internet of things (IoT) for safety and efficiency in construction building site operations
Source: Sci Rep. 2024 Nov 21;14:28914. doi: 10.1038/s41598-024-78931-0 (PMC11582823; doi:10.1038/s41598-024-78931-0)
Supplement: Supplementary file 1 — Supplementary Material 1 [file 41598_2024_78931_MOESM1_ESM.docx]

# Survey Questionnaire

**Research Title:** **Internet of Things (IoT) for Safety and Efficiency in Construction Building Site Operations**

1. **What is your profession?**
   - Quantity Surveyor
   - Architect
   - Civil Engineer
   - M&E Engineer
   - Project Manager
   - Other
2. **What is your organization?**
   - Contractor
   - Consultant
   - Client
3. **What is your experience in Malaysia's construction industry ?**
   - 0-5 Years
   - 6-10 Years
   - 11-15 Years
   - 16-20 Years
   - Over 20 Years
4. **What is your current knowledge of BIM?**
   - Yes
   - No/Maybe

| **Sr. #** | **Internet of Things (IoT) for Safety and Efficiency in Construction Building Site Operations** | **Strongly Disagree (1)** | **Disagree (2)** | **Neutral (3)** | **Agree (4)** | **Strongly Agree (5)** |
| --- | --- | --- | --- | --- | --- | --- |
| 1 | Sensors of the Internet of Things collect data in real-time on equipment performance, usage patterns, and operating conditions. |  |  |  |  |  |
| 2 | Optimal maintenance planning increases equipment dependability and longevity. |  |  |  |  |  |
| 3 | Early defect or malfunction detection enables proactive maintenance to minimize downtime. |  |  |  |  |  |
| 4 | Real-time surveillance ensures compliance with safety regulations and promotes a healthier workplace. |  |  |  |  |  |
| 5 | Predictive maintenance models facilitate proactive maintenance scheduling, thereby minimizing unscheduled delays. |  |  |  |  |  |
| 6 | Data on fuel consumption, engine heath, and operating conditions facilitates equipment utilization optimization. |  |  |  |  |  |
| 7 | IoT-based wearable devices can detect falls or incidents and automatically activate distress signals for prompt assistance. |  |  |  |  |  |
| 8 | Environmental monitoring improves site-wide security, worker health, and sustainability. |  |  |  |  |  |
| 9 | In high-risk areas, Internet of Things sensors can detect hazardous conditions and alert workers and managers to prevent accidents. |  |  |  |  |  |
| 10 | Tracking the location of employees in real-time ensures their safety and enables effective emergency response. |  |  |  |  |  |
| 11 | Internet of Things sensors measure environmental parameters such as air quality, noise levels, temperature, and humidity. |  |  |  |  |  |
| 12 | Wearable devices facilitated by the Internet of Things, such as smart headgear and safety garments, monitor employees' vital signs and fatigue levels in real time. |  |  |  |  |  |
| 13 | Enhanced equipment management increases productivity and reduces expenses. |  |  |  |  |  |
| 14 | Insights derived from data improve resource allocation and optimize construction processes. |  |  |  |  |  |
| 15 | IoT systems allow for the management and optimization of energy consumption. |  |  |  |  |  |
| 16 | In construction locations, predictive analytics improve operational efficiency and productivity. |  |  |  |  |  |
